# Supplementary material for: Design and Characterization of a Novel Hapten and Preparation of Monoclonal Antibody for Detecting Atrazine
Source: Foods. 2022 Jun 13;11(12):1726. doi: 10.3390/foods11121726 (PMC9223028; doi:10.3390/foods11121726)
Supplement: Supplementary file 1 [file foods-11-01726-s001.zip › foods-1741655-supplementary.pdf]

Design and characterization of a novel hapten and preparation of monoclonal antibody for detecting atrazine

Lingyuan Xu [a], A.M. Abd El-Aty[b,c], Jae-Han Shim[d], Jong-Bang Eun[e], Xingmei Lei [a], Jing Zhao [a], Xiuyuan Zhang [a], Xueyan Cui [a], Yongxin She [a], Fen Jin[a], Lufei Zheng[a], Jing Wang[a], Maojun Jina * and Bruce D. Hammock[f]

[a] *Institute of Quality Standard and Testing Technology for Agro-Products, Chinese Academy of Agricultural Sciences, Beijing 100081, China*

[b] *Department of Pharmacology, Faculty of Veterinary Medicine, Cairo University, Giza 12211, Egypt*

[c] *Department of Medical Pharmacology, Medical Faculty, Ataturk University, Erzurum 25240, Turkey*

[d] *Natural Products Chemistry Laboratory, Biotechnology Research Institute, Chonnam National University, Yongbong-ro, Buk-gu, Gwangju 500-757, Republic of Korea*

[e] *Department of Food Science and Technology, Chonnam National University, Gwangju, South Korea*

[f] *Department of Entomology & Nematology and the UC Davis Comprehensive Cancer Center, University of California, Davis, CA 95616, USA*

*** Corresponding authors**

Maojun Jin, **E-mail**: jinmaojun@caas.cn, **Tel.**: +86-10-8210-6570

Table S1. Serum potency and inhibition rate of ATZ in mice.

Immunization cycle	Dilution of immunogen (Serum dilution)	0.5×10^3			1×10^3			2×10^3			4×10^3		
		C	I	IR	C	I	IR	C	I	IR	C	I	IR
**Third immunization**	0.5×10^3	3.5136	3.5654	−1.47%	3.5004	3.6343	-3.83%	3.5473	3.6033	−1.58%	3.3066	3.3732	−2.01%
	1×10^3	3.6391	3.4826	4.30%	3.6913	3.4873	5.53%	3.5664	3.4228	4.03%	3.6266	3.0506	15.88%
	2×10^3	3.4913	3.3666	3.57%	3.6095	3.3089	8.33%	3.4381	2.9646	13.77%	3.3302	2.7037	18.81%
	4×10^3	3.4086	3.1808	6.68%	3.2329	2.9949	7.36%	3.3495	2.5193	24.79%	3.2076	1.927	39.92%
**Immunization cycle**	**Dilution of immunogen (Serum dilution)**	2×10^3			4×10^3			8×10^3			16×10^3		
		C	I	IR	C	I	IR	C	I	IR	C	I	IR
**Fourth immunization**	8×10^3	3.8307	3.0001	21.68%	3.7991	2.7415	27.84%	3.5665	2.3499	34.11%	3.6115	1.7387	51.86%
	16×10^3	3.6223	1.5249	57.90%	3.6875	1.5868	56.97%	3.5247	1.2492	64.56%	3.2604	0.8371	74.33%
	32×10^3	2.9148	0.7982	72.62%	2.8312	0.8484	70.03%	2.3959	0.4884	79.62%	2.0832	0.3863	81.46%
	64×10^3	1.3083	0.5222	60.09%	2.8312	0.8484	70.03%	1.2363	0.2959	76.07%	1.0204	0.2574	74.77%

Note: "C" represents control wells, "I" represents inhibition wells, and "IR" represents inhibition rate. The inhibitory concentration of ATR was 100 ng/mL.

Table S2. Serum titer and inhibition rate of mouse for fusion experiment.

Dilution of immunogen (Serum dilution times)	2×10^3			4×10^3			8×10^3			16×10^3		
	C	I	IR	C	I	IR	C	I	IR	C	I	IR
**0.5K**	2.951	3.1162	−5.60%	3.13	3.2666	−4.36%	3.2514	3.4754	−6.89%	3.4926	3.5829	−2.59%
**1K**	3.0572	3.0072	1.64%	3.0814	3.2014	−3.89%	3.1114	3.2247	−3.64%	3.2167	3.1608	1.74%
**2K**	3.0257	3.0787	−1.75%	3.1605	3.3588	−6.27%	3.2629	3.4687	−6.31%	3.1772	3.1995	−0.70%
**4K**	3.0563	3.0167	1.30%	2.7498	3.151	−14.59%	3.12	3.296	−5.64%	3.2234	3.0907	4.12%
**8K**	3.0067	3.0637	−1.90%	2.7363	3.5006	−27.93%	3.0554	3.4181	−11.87%	3.136	2.5625	18.29%
**16K**	3.0408	3.272	−7.60%	2.8192	3.3036	−17.18%	3.213	2.6117	18.71%	3.3254	1.6754	49.62%
**32K**	3.234	2.7661	14.47%	3.3175	2.1717	34.54%	3.3523	1.3781	58.89%	3.4339	0.8096	76.42%
**64K**	3.2949	2.6451	19.72%	3.3516	1.6636	50.36%	3.1482	0.8823	71.97%	2.7227	0.5444	80.01%
**Dilution of immunogen (Serum dilution times)**	**32×10^3**			**64×10^3**			**128×10^3**			**256×10^3**		
	**C**	**I**	**IR**	**C**	**I**	**IR**	**C**	**I**	**IR**	**C**	**I**	**IR**
**0.5K**	3.4679	3.1281	9.80%	3.472	3.5448	−2.10%	1.4202	0.8769	38.26%	0.7161	0.7447	−3.99%
**1K**	3.3136	3.6357	−9.72%	3.0714	3.0347	1.19%	1.2275	0.816	33.52%	0.6642	0.3648	45.08%
**2K**	3.4255	3.3286	2.83%	3.5234	2.6797	23.95%	1.0295	0.3413	66.85%	0.6154	0.2071	66.35%
**4K**	3.3286	2.696	19.00%	3.497	1.7998	48.53%	0.8119	0.2032	74.97%	0.5308	0.1493	71.87%
**8K**	3.4857	1.8158	47.91%	3.4263	1.0965	68.00%	0.6297	0.166	73.64%	0.4437	0.122	72.50%
**16K**	3.4798	0.9567	72.51%	3.1996	0.6055	81.08%	0.4244	0.1259	70.33%	0.2879	0.1195	58.49%
**32K**	3.0233	0.4523	85.04%	2.2536	0.3176	85.91%	0.2812	0.1172	58.32%	0.1931	0.1526	20.97%
**64K**	1.993	0.3236	83.76%	1.4006	0.222	84.15%	0.2388	0.1477	38.15%	0.2111	0.2275	−7.77%

Note: “C” represents control wells, “I” represents inhibition wells, and “IR” represents inhibition rate. Inclusion source concentration 1 mg/mL. The inhibitory concentration of ATR was 1000 ng/mL.

Table S3. Titer and inhibition rate of mouse ascites before purification.

Dilution of immunogen	**1.6×10^4**			**3.2×10^4**		
(Serum dilution times)	**C**	**I**	**IR**	**C**	**I**	**IR**
**16K**	Overflow	0.1467	—	3.9433	0.1259	96.81%
**32K**	Overflow	0.1181	—	3.859	0.094	97.56%
**64K**	3.8019	0.1776	95.33%	3.4209	0.0917	97.32%
**128K**	3.0072	0.1009	96.64%	2.6763	0.0965	96.39%
**256K**	2.112	0.1159	94.51%	1.7206	0.0957	94.44%
Dilution of immunogen	**6.4×10^4**			**1.28×10^5**		
(Serum dilution times)	**C**	**I**	**IR**	**C**	**I**	**IR**
**16K**	3.6164	0.0981	97.29%	2.5838	0.0964	96.27%
**32K**	3.2234	0.0858	97.34%	2.027	0.0853	95.79%
**64K**	2.3793	0.0837	96.48%	1.571	0.0835	94.68%
**128K**	1.7889	0.0879	95.09%	1.0773	0.1127	89.54%
**256K**	1.1408	0.0914	91.99%	0.6757	0.0988	85.38%

Note: "C" represents control wells, "I" represents inhibition wells, and "IR" represents inhibition rate. The concentration of the coating agent was 1 mg/mL. The inhibitory concentration of ATR was 100 ng/mL. "Overflow" indicated that the $OD450_{nm}$ value was outside the detection range of the instrument. "—" indicates that the result are not calculated.

Table S4. Determination of the optimal concentrations of antigen and antibody.

Dilution of immunogen	**2×10³**			**4×10³**			**8×10³**			**16×10³**		
(Serum dilution times)	**C**	**I**	**IR**	**C**	**I**	**IR**	**C**	**I**	**IR**	**C**	**I**	**IR**
**2K**	3.6308	2.368	34.78%	3.5449	1.1545	67.43%	3.8403	0.5585	85.46%	3.4926	3.5829	−2.59%
**4K**	3.6817	1.2912	64.93%	3.6933	0.64	82.67%	3.7294	0.2896	92.23%	3.2167	3.1608	1.74%
**8K**	3.756	0.7204	80.82%	3.5538	0.3863	89.13%	3.3562	0.1906	94.32%	3.1772	3.1995	−0.70%
**16K**	3.1678	0.4306	86.41%	3.4571	0.2385	93.10%	3.103	0.145	95.33%	3.2234	3.0907	4.12%
**32K**	2.715	0.2321	91.45%	2.2273	0.1489	93.31%	1.9538	0.1086	94.44%	3.136	2.5625	18.29%
**64K**	1.547	0.1704	88.99%	1.3833	0.1177	91.49%	1.158	0.0967	91.65%	3.3254	1.6754	49.62%
**128K**	0.8689	0.1238	85.75%	0.7856	0.1007	87.18%	0.7009	0.0837	88.06%	3.4339	0.8096	76.42%
**256K**	0.5411	0.1105	79.58%	0.4537	0.0889	80.41%	0.3985	0.0848	78.72%	2.7227	0.5444	80.01%
Dilution of immunogen	**32×10³**			**64×10³**			**128×10³**			**256×10³**		
(Serum dilution times)	**C**	**I**	**IR**	**C**	**I**	**IR**	**C**	**I**	**IR**	**C**	**I**	**IR**
**2K**	3.6581	0.297	91.88%	2.9236	0.2119	92.75%	1.9484	0.1522	92.19%	0.7161	0.7447	**-3.99%**
**4K**	3.1739	0.177	94.42%	2.6811	0.1347	94.98%	1.7007	0.1129	93.36%	0.6642	0.3648	45.08%
**8K**	2.9019	0.125	95.69%	1.9539	0.1089	94.43%	1.2453	0.0975	92.17%	0.6154	0.2071	66.35%
**16K**	2.1751	0.1029	95.27%	1.4851	0.0937	93.69%	0.9228	0.0841	90.89%	0.5308	0.1493	71.87%
**32K**	1.368	0.0865	93.68%	0.8325	0.0829	90.04%	0.5497	0.0852	84.50%	0.4437	0.122	72.50%
**64K**	0.8175	0.0804	90.17%	0.5236	0.0808	84.57%	0.3527	0.0804	77.20%	0.2879	0.1195	58.49%
**128K**	0.4996	0.0808	83.83%	0.361	0.0764	78.84%	0.2353	0.0773	67.15%	0.1931	0.1526	20.97%
**256K**	0.2966	0.0781	73.67%	0.2135	0.0774	63.75%	0.156	0.0739	52.63%			

Note: "C" represents control wells, "I" represents inhibition wells, and "IR" represents inhibition rate. The inhibitory concentration of ATR was 100 ng/mL.

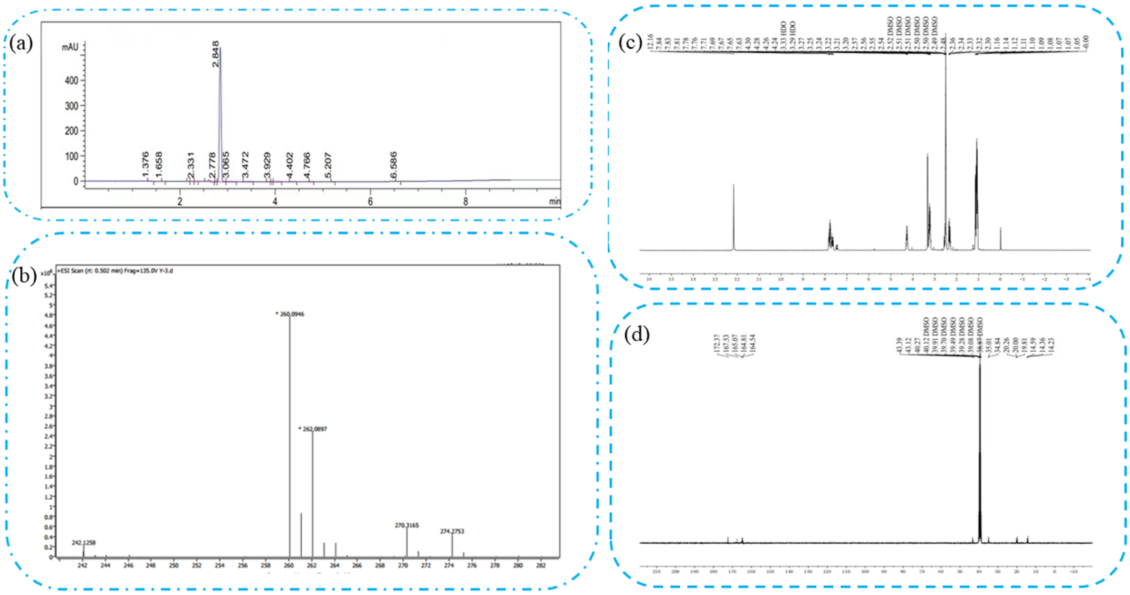

Figure S1. Identification of ATR hapten using HPLC (**a**), HRMS (**b**), 1H NMR (**c**), and 13C NMR (**d**) analysis.

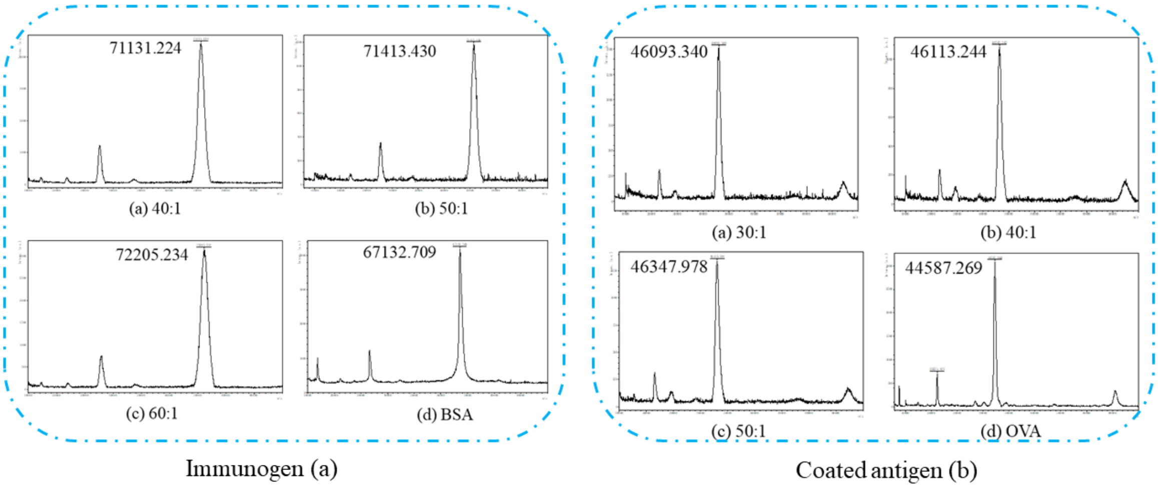

Figure S2. MALDI-TOF-MS results of the immunogen (**a**) and coated antigen (**b**)
